# Presumed Choroidopathy of IgG4-Related Disease Discovered During 16-Year Follow-Up of a Patient With Polycystic Kidney Disease

**DOI:** 10.7759/cureus.70865

**Published:** 2024-10-04

**Authors:** Toshihiko Matsuo, Takehiro Tanaka, Kenji Tsuji

**Affiliations:** 1 Ophthalmology, Graduate School of Interdisciplinary Science and Engineering in Health Systems, Okayama University, Okayama, JPN; 2 Ophthalmology, Okayama University Hospital, Okayama, JPN; 3 Pathology, Okayama University Hospital, Okayama, JPN; 4 Nephrology, Okayama University Hospital, Okayama City, JPN

**Keywords:** acute posterior multifocal placoid pigment epitheliopathy, choroidopathy, igg4-related disease, lacrimal gland tumor, uveitis

## Abstract

Immunoglobulin G4 (IgG4)-related disease is characterized by infiltration with IgG4-producing plasma cells in different organs and the elevation of serum IgG4. We present a patient with polycystic kidney disease in long-term follow-up who developed bilateral lacrimal gland enlargement and presumed IgG4-related choroidopathy at different time points. A 45-year-old woman developed bilateral upper eyelid swelling. Head MRI showed bilateral lacrimal gland enlargement, and the resection on both sides revealed foci of infiltration with lymphocytes and plasma cells in bilateral lacrimal glands. The IgG4-immunostaining did not satisfy the diagnostic criteria. She had been taking oral valsartan 40 mg daily for hypertension with polycystic kidney disease.

The patient was well until the age of 49 years, when she noticed yellowish vision in the right eye compared to the left eye. The right eye showed multiple yellowish spotty lesions in the deep retina to choroid with a mildly hyperemic optic disc, while the left eye showed the normal fundus. No inflammation was noted in the anterior segments of both eyes. Fundus angiography demonstrated early-phase no-filling with late-phase leakage by fluorescein dye and both early-phase and late-phase no-filling by indocyanine green dye, leading to the diagnosis of acute posterior multifocal placoid pigment epitheliopathy (APMPPE). She began to have oral prednisolone tapered from 30 mg daily and discontinued in a year. At the age of 52 years, she switched to candesartan 8 mg daily and began to have tolvaptan (a selective competitive vasopressin receptor 2 (V_2_) antagonist) 90 mg daily for polycystic kidney disease with liver cysts. At that time, the lesions in the right eye had mild degeneration.

The patient was followed once a year ophthalmologically to maintain good vision. At 57 years, serum IgG4, which was measured for the first time on suspicion of IgG4-related disease, was elevated to 269.6 mg/dL. In the following four years to the latest visit at 61 years, she kept stable but high levels of serum IgG4 around 300 mg/dL. Serum IgG4 measurement is helpful to make a clinical diagnosis and, hence, a clinical decision since the spectrum of IgG4-related disease remains obscure.

## Introduction

Immunoglobulin G4 (IgG4)-related disease is precipitated by infiltration with IgG4-producing plasma cells as well as fibrotic changes in different organs [1]. The diagnosis of IgG4-related disease is based pathologically on IgG4 immunostaining of excised tissue or biopsy specimen [2]. The comprehensive diagnostic criteria for IgG4-related disease were established in 2012 [3], and since then, the measurement of serum IgG4 has been covered by reimbursement of national health insurance in Japan. To reach a consensus worldwide, the 2019 classification criteria for IgG4-related disease has been published [4]. Watchful waiting and no therapy is justified when the patient with IgG4-related disease has no symptoms. Appropriate corticosteroid administration is a treatment recommendation in the case that the disease is causing symptoms [3,4].

Ophthalmologically, lacrimal glands are frequent sites of involvement with IgG4-related disease [5,6]. Recently, IgG4-related disease is suggested to be considered in differential diagnoses for non-infectious uveitis [7,8]. Uveitis, in general, indicates intraocular tissue inflammation caused by both infectious and non-infectious different kinds of diseases [9,10]. As one entity of non-infectious uveitis, acute posterior multifocal placoid pigment epitheliopathy (APMPPE) is characterized by multifocal deep retinochoroidal yellowish spotty lesions and is sometimes associated with antineutrophil cytoplasmic antibody (ANCA)-associated vasculitis [9,10]. This study presents a patient with APMPPE in the background of a high serum level of IgG4.

## Case presentation

**Figure 1:**
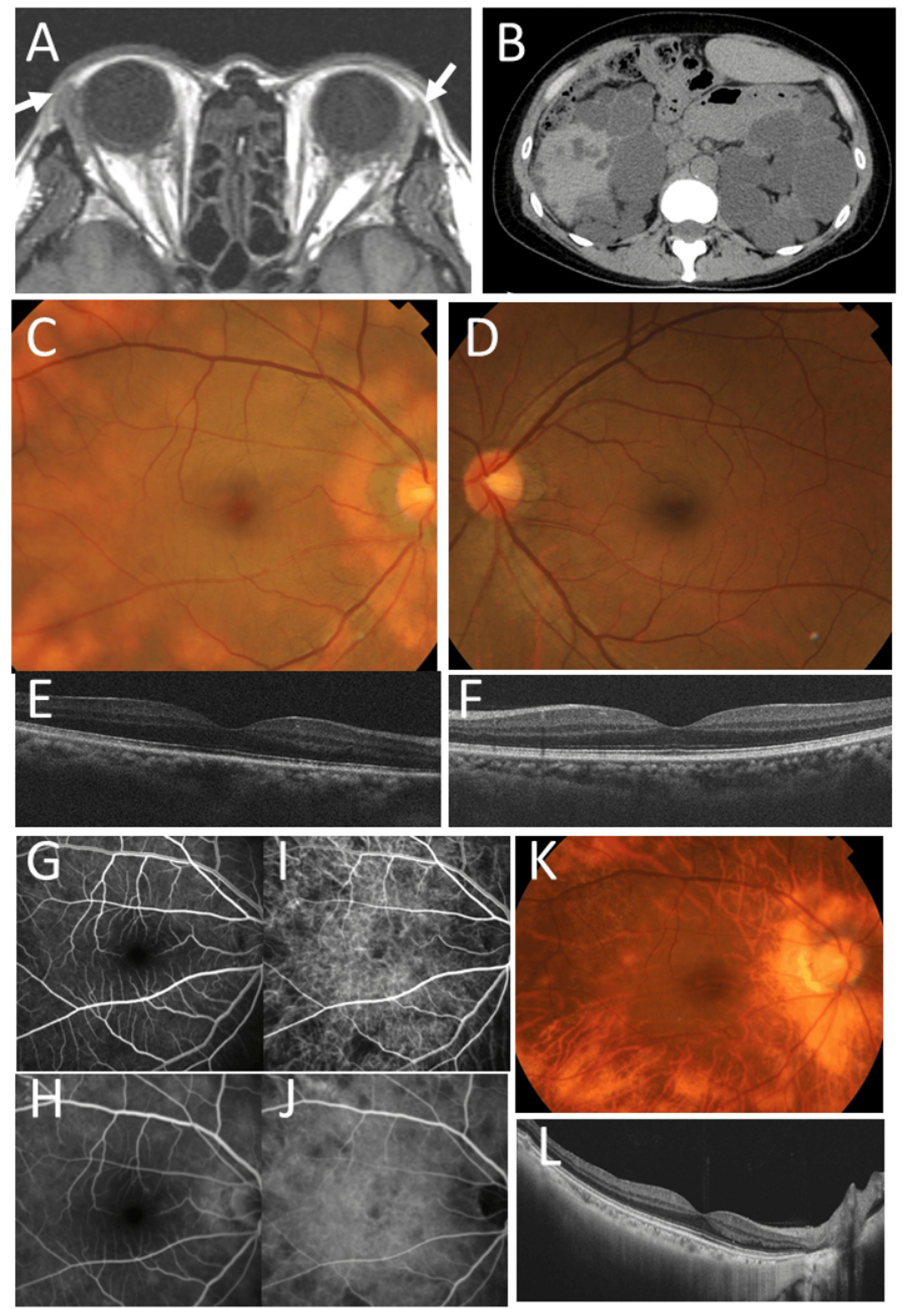
MRI, CT scan, and fundus images The MRI shows bilateral lacrimal gland enlargement at the age of 45 years (arrows; A). The CT scan shows bilateral polycystic kidneys at the age of 53 years (B). At the age of 49 years, fundus photographs (C) show yellowish spotty deep retinal to choroidal lesions in the right eye and normal fundus in the left eye (D). The optical coherence tomography in the horizontal section appears normal in the right eye (E) and left eye (F). The fundus angiography in the right eye shows early-phase no filling (G) and late leakage (H) with fluorescein dye and both early-phase (I) and late-phase (J) no filling with indocyanine green. At the age of 57 years, the fundus photograph shows mild degenerative retinochoroidal changes (K), while the optical coherence tomography reveals mild thinning of the choroid (L) in the right eye.

**Figure 2:**
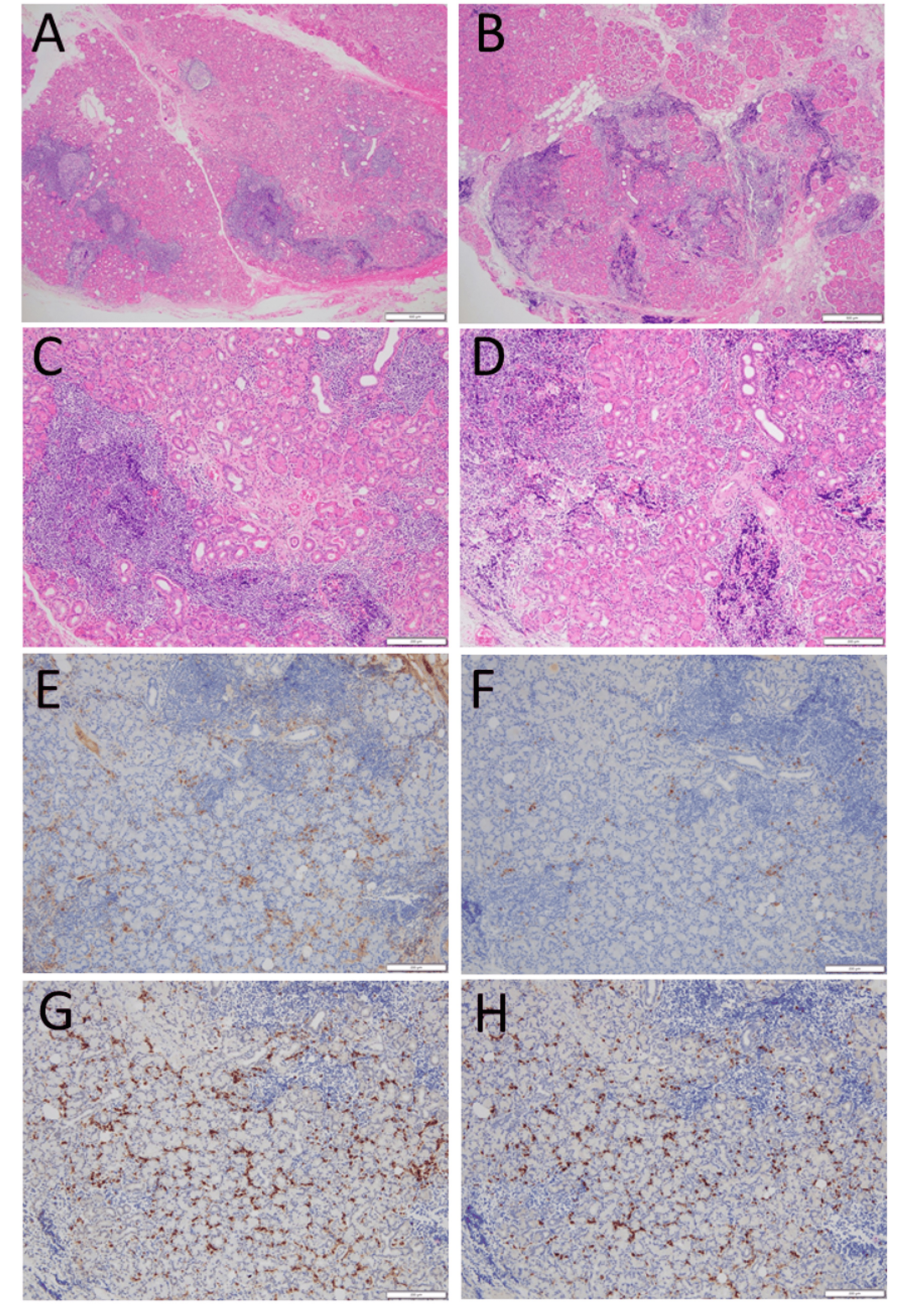
Pathology of lacrimal glands Seen are foci of infiltration with lymphocytes and plasma cells among lacrimal gland acini on the right side (low magnification in A, high magnification in C) and left side (low magnification in B, high magnification in D) via hematoxylin-eosin stain. A small number of IgG-positive cells (E) and IgG4-positive cells (F) are identified by immunohistochemistry on the left side. Immunoglobulin light chain κ-positive cells (G) and λ-positive cells (H) are seen in equal distribution by in situ hybridization on the left side. Immunostaining on the right side (not shown) appeared the same as on the left side. White scale bar = 500 µm in A and B and 200 µm in C-H

The patient is a 45-year-old woman who developed bilateral upper eyelid swelling. The MRI of the head showed bilateral lacrimal gland enlargement (Figure 1A) and the resection on both sides revealed foci of infiltration with lymphocytes and plasma cells in bilateral lacrimal glands (Figure 2 A-D). However, the IgG4-immunostaining did not satisfy the diagnostic criteria (Figure 2 E-H). Since the resection relieved her symptoms, she had no additional treatment, including corticosteroid administration.

The patient was on oral valsartan 40 mg daily for hypertension with polycystic kidney disease. She was well until the age of 49 years when she noticed yellowish vision in the right eye when compared with the left eye. The best-corrected visual acuity in decimals was 1.5 in both eyes. The right eye showed multiple yellowish spotty lesions in the deep retina to choroid with mildly hyperemic optic disc (Figure 1C and Figure 1E) while the left eye showed the normal fundus (Figure 1D and Figure 1F). There was no inflammation in the anterior segments of both eyes. The fundus angiography demonstrated early-phase no-filling with late-phase leakage by fluorescein dye (Figure 1G and Figure 1H) and both early-phase and late-phase no-filling by indocyanine green dye (Figure 1I and Figure 1J). Thus, we arrived at APMPPE as the diagnosis.

Following this, the patient's oral prednisolone was tapered from 30 mg daily and discontinued in a year. At the age of 52, she switched to candesartan 8 mg daily and started on tolvaptan (a selective competitive vasopressin receptor 2 (V2) antagonist) 90 mg daily for polycystic kidney disease (Figure 1B) with liver cysts. At that time, the estimated glomerular filtration rate (eGFR) was 52.4 mL/min/1.73 m2 (normal range: ≥ 60); the lesions in the right eye had mild degeneration (Figure 1K and Figure 1L).

The patient had an annual ophthalmological follow-up to maintain good vision. At 57 years, her serum IgG4 was measured for the first time on suspicion of IgG4-related disease; it was elevated to 269.6 mg/dL (normal range: 14.6-117). In the following four years, i.e., at 61 years, she maintains a stable but high level of serum IgG4 (around 300 mg/dL). The eGFR decreased to 37.8 mL/min/1.73 m2. At the latest visit, the best-corrected visual acuity in decimals was 1.5 in both eyes. The intraocular pressure was 16 mmHg in both eyes. She showed no cataracts and used no topical medications. 

## Discussion

The present patient is unique due to the development of bilateral lacrimal gland enlargement, APMPPE in the right eye, and persistent elevation of serum IgG4 levels in the 16-year follow-up period of polycystic kidney disease. In other words, these events, which would otherwise be considered unrelated with one another, could be documented because she was followed semiannually for polycystic kidney disease. It is natural to think that bilateral lacrimal gland enlargement, APMPPE, and serum IgG4 elevation would be somewhat related to one another. In contrast, polycystic kidney disease is a genetic disorder and not of inflammatory origin, and thus is considered unrelated to these manifestations.

A major limitation in this patient is that the timing of the events was not simultaneous: she first developed bilateral lacrimal gland enlargement, and the pathology did not satisfy the criteria for IgG4-related disease. At that time, serum IgG4 measurement was not available clinically. She then developed APMPPE and had oral prednisolone tapering in a year. Concurrently, she had polycystic kidney disease and was followed with tolvaptan and candesartan prescribed by a nephrologist. In this process, serum IgG4 was measured to show consistently high values. Based on these events, she was estimated to develop lacrimal gland enlargement and APMPPE in the background of IgG4-related disease. The lacrimal gland lesions at the resection might be in too early a phase to show sufficient number of IgG4-positive cells. The IgG4-related kidney disease [11,12] might be concurrently present in this patient, but renal biopsy to prove the IgG4-related condition was not indicated in polycystic kidney disease.

The APMPPE appears to have obliterative inflammation of lobular vasculature of choriocapillaris in the choroid, as evidenced by early-phase no-filling in fundus angiography. The vascular inflammation in the choroid is also supported by the fact that APMPPE occurs in ANCA-associated vasculitis [9,10]. The IgG4-related vasculitis has also been known as an entity that may sometimes be coexistent with ANCA-associated vasculitis [13,14]. The ANCA might belong to an IgG4 subclass [13]. Under the circumstances, serum IgG4 as well as MPO-ANCA and PR3-ANCA should be measured in patients with uveitis, especially APMPPE. The IgG4-related disease has been reported to develop choroidal mass lesions which mimic choroidal tumors such as lymphoma, leading to inadvertent enucleation in some cases [15-17]. Inflammatory choroidal neovascularization was also documented in association with high serum IgG4 [18]. Serum IgG4 measurement would become a diagnostic aid on these occasions. Furthermore, the patient should be followed carefully in the long term since IgG4-related disease and lymphoma would serve as the background for reciprocal development of each disease [19,20].

## Conclusions

Our patient in the 16-year follow-up period for polycystic kidney disease developed bilateral lacrimal gland enlargement and APMPPE in the background of elevated serum IgG4 levels. The APMPPE, an entity of non-infectious uveitis that is derived from choroidal vascular inflammation, is presumed to be IgG4-related in this patient. Serum IgG4 measurement helps make a clinical diagnosis and, hence, a clinical decision, especially since the spectrum of IgG4-related disease still remains obscure. The long-term follow-up may give insight into the pathogenesis of the disease now that a new clinical test such as serum IgG4 measurement has become available in recent decades.
